# Mechanism-Based Pharmacokinetic/Pharmacodynamic Modeling of Erythroferrone in Anemic Rats with Chronic Kidney Disease and Chemotherapy-Induced Anemia: An Early Biomarker for Hemoglobin Response and rHuEPO Hyporesponsiveness

**DOI:** 10.1021/acsptsci.4c00575

**Published:** 2024-12-11

**Authors:** Lin Zhang, Peng Xu, Xiaoyu Yan

**Affiliations:** Guangdong-Hong Kong-Macao Joint Laboratory for New Drug Screening, School of Pharmacy, The Chinese University of Hong Kong, Shatin 999077, Hong Kong SAR, P. R. China

**Keywords:** erythroferrone, chemotherapy-induced anemia, chronic kidney disease-associated anemia, rHuEPO hyporesponsiveness, mechanism-based PK/PD model

## Abstract

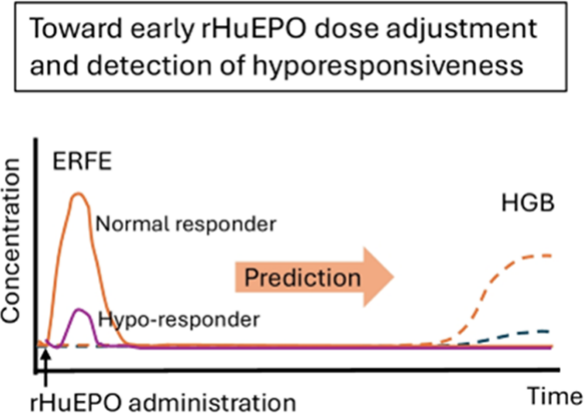

Erythroferrone (ERFE) has emerged as a potential biomarker for the erythropoiesis response following recombinant human erythropoietin (rHuEPO) treatment. While the association between ERFE and hemoglobin (HGB) response to rHuEPO is well-established in nonanemic conditions, such correlation and ERFE kinetics in anemic states remain unclear. We employed two rat models of anemia, chronic kidney disease (CKD) anemia and chemotherapy-induced anemia (CIA), to determine ERFE kinetics and its correlation with HGB responses after rHuEPO administration. The key factors influencing ERFE kinetics were characterized using a PK/PD modeling approach and supported by experimentation. Following rHuEPO injection, ERFE induction was diminished in anemic rats compared with that of healthy rats, primarily attributed to the reduced precursor cell mass and impaired rHuEPO responsiveness. The early increase in ERFE at 4 h post administration allows for the prompt prediction of HGB response and rHuEPO hyporesponsiveness in anemic rats. Consequently, the ERFE-based dose adjustment resulted in a rHuEPO-sparing effect in CKD rats. This strategy is expected to be translatable to anemic patients, potentially reducing rHuEPO doses and mitigating HGB overshooting.

Anemia is a common side effect among patients with chronic kidney disease (CKD) or receiving chemotherapy, also termed chemotherapy-induced anemia (CIA). The prevalence of anemia was 64% in CKD patients^1^ and as high as 90% among patients receiving chemotherapy.^2^ Emerging studies demonstrated that anemia negatively influenced the quality of life and increased the incidence of cardiovascular disease and mortality among these patients.^3,4^ The mechanisms by which CKD and chemotherapy cause anemia are multifactorial. In CKD, anemia is primarily caused by relative impaired erythropoietin (EPO) production and/or EPO responsiveness.^5^ CIA, on the other hand, is mainly caused by myelosuppression.^6^

Recombinant human erythropoietin (rHuEPO) is a type of erythropoiesis-stimulating agent (ESAs) that has been approved to treat anemia in CKD patients and CIA in cancer patients with hemoglobin (HGB) at 10g/dL or below. In clinical practice, rHuEPO is initiated based on HGB and individualized by HGB increasing rate to achieve a target HGB level to minimize adverse effects while avoiding blood transfusion. However, it usually takes 4 weeks (minimum of 2 weeks) to show a significant increase in HGB response after rHuEPO treatment. Importantly, some patients exhibit inadequate or poor response to EPO, which is associated with higher mortality rate.^7,8^ A retrospective cohort study found that approximately 20% of CKD and cancer patients receiving ESA treatment were hyporesponders, exhibiting no change or a decrease in HGB levels within the initial 3 months of ESA therapy.^9^ The cause of hyporesponsiveness to rHuEPO is multifactorial, involving factors such as inflammation-induced enhanced hepcidin, which disrupts iron metabolism, promoted apoptosis of erythroid progenitor cells, sequestration of iron in macrophages, malignancy, and myelosuppressive drugs.^10^ Additionally, excessive rHuEPO dosages and overshooting of HGB are associated with increased risks of thrombotic events, cardiovascular complications, and higher mortality.^11,12^ Therefore, an early biomarker for HGB response and rHuEPO hyporesponsiveness is urgently needed to facilitate the early dose titration of rHuEPO and identify hyporesponders promptly. This approach could prevent potential harm resulting from rHuEPO dose escalation and improve patient outcomes. Currently, ESA resistance index (ERI) is an available predictor for rHuEPO resistance, but its validity is controversial.^13,14^ Serum albumin^15^ and baseline hepcidin have been reported to be associated with HGB response in patients following rHuEPO therapy.^16^ However, these predictors are not yet recommended for use in the clinical setting.

Erythroferrone (ERFE), a hormone belonging to the family of tumor necrosis factor-α, is secreted mainly by erythroblasts and serves as a signal that communicates the iron demand under stress erythropoiesis. ERFE suppresses hepcidin expression by inhibiting the BMP-SMAD pathway,^17,18^ thereby allowing the stabilization of iron exporter ferroprotein and enhancing iron utilization for erythropoiesis. Analogous to using the molecules secreted by tumor cells to predict long-term survival among cancer patients,^19^ we propose that the ERFE response might serve as an early biomarker in predicting long-term HGB response to rHuEPO in anemic patients. Indeed, ERFE increases at very low rHuEPO levels (20 IU/kg) in healthy subjects.^20^ And the ERFE level increases as early as 2 h after the injection of rHuEPO in murine models.^17,21^

So far, studies evaluating the predictive value of ERFE have focused on its relationship with serum erythropoietin. Clinical studies have reported a positive correlation between ERFE and erythropoietin levels in healthy subjects and CKD patients, revealing its application in blood doping and altitude training.^20,22,23^ Only some studies reported the correlation between ERFE and HGB, however, with contradictory results. A positive correlation between peak concentrations of ERFE and HGB after receiving rHuEPO treatment in healthy rats was reported,^21,24^ whereas a clinical study revealed a negative correlation between baseline ERFE and HGB in anemic patients.^25^ Notably, in anemic patients, the baseline ERFE is upregulated.^25−27^ Given that the peak ERFE does not solely reflect the ERFE released following rHuEPO treatment as it is influenced by its baseline levels and its kinetics, the association between ERFE and HGB responses in anemic states remains unknown. So, we investigated the ERFE kinetics and its correlation with the HGB response following rHuEPO treatment in two well-established anemic rat models (i.e., adenine-induced CKD and carboplatin-induced CIA). We further employed a PK/PD model to quantify the impact of pathological factors on ERFE kinetics, thereby supporting its utility as an early biomarker. This study involved two main objectives: (1) characterizing ERFE kinetics in anemic rats and identification of its key determinants and (2) exploring the utility of ERFE response as a predictor for HGB response and rHuEPO hyporesponsiveness in anemic rats.

## Methods

### Animal Studies

All animal studies were conducted with the approval of the Animal Experimentation Ethics Committee of The Chinese University of Hong Kong (Reference Number 20–301-MIS). All experiments were performed using male Sprague–Dawley rats weighing from 250 to 300 g provided by the Laboratory Animal Services Centre at The Chinese University of Hong Kong. Details on the experimental procedures performed in CKD rats and CIA rats are provided in the Supporting Information (Figure S1).

#### CKD Rats

CKD-associated anemia was induced as described elsewhere.^28^ Briefly, rats received adenine (Sigma-Aldrich, 600 mg/kg, once a day) by gavage for 6 days, followed by a reduced dose (300 mg/kg once a day) for 6 days, and stabilized for 1 week. Rats were kept on a standard food diet supplemented with 1% (w/w) carbonyl iron (test diet). After 3 weeks (“Week 0”), CKD rats were randomly allocated into treatment groups receiving recombinant human erythropoietin (HuEPO, EPOGEN 20,000 IU/ml, Amgen Inc., Thousand Oaks, CA) at 1350 IU/kg (*n* = 6) or 450 IU/kg (*n* = 6) t.i.w. or nontreatment group receiving saline (*n* = 5) for 2 weeks (all given i.v.). A rotation sampling method was used to minimize the impact of blood loss on the quantification of ERFE. ERFE assay was conducted at 0, 1, 2, 4, 6, 8, 10, 12, and 24 h after injection of HuEPO or saline. Blood samples (100–150 uL) were collected via tail vein on “Week 0” days 0, 4, 10, 15, 20, 25, 30, and 34 for hematological analysis.

#### CIA Rats

CIA was induced by injecting a single dose of carboplatin at 60 mg/kg (MedChemExpress) via the tail vein. The control group received saline only. At 1 week after carboplatin (“Week 0”), CIA rats were randomly allocated into treatment groups receiving a single dose of rHuEPO at 1350 IU/kg or 450 IU/kg or nontreatment group (*n* = 9 per group) receiving saline only (all given i.v.). A rotation sampling method was used to minimize the impact of blood loss on quantification of ERFE. ERFE assay was conducted at 0, 1, 2, 4, 6, 8, 10, 12, and 24 h after the injection of HuEPO or saline. Blood samples (100–150 μL) were collected via tail vein on “Week −1” days −8, −6, −4, −2, 0, 3, 6, 9, 12, 13, 14, 16, 17, 18, 20, 21, and 24 for hematological analysis. Rats (*n* = 3 each group per day) were sacrificed for the sampling of bone marrow and spleen on 0, 1, 4, 8, 11, 14, and 15 days after carboplatin or saline treatment for flow cytometry analysis.

#### rHuEPO-Sparing Study in CKD Rat

CKD rats with HGB levels below 13 g/dL were included as CKD anemic rats. Details of study design, including dosing and sampling schedules and rHuEPO dose titration rules, are provided in the Supporting Information (Figure S2). To mimic rHuEPO usage in clinical situations, CKD rats were dosed with an initial dose of rHuEPO at 100 IU/kg (tiw, i.v. injection) in “Week 1” and individualized for the maintenance period (Week 2 and Week 3). Specifically, for HGB-based dosing, the rHuEPO dose is increased by 25% if the change of HGB within 5 days dropped below 2 g/dL or else the rHuEPO dose is reduced by 25%. For ERFE-based dosing, the rHuEPO dose is increased by 25% if the change of ERFE at 4 h from baseline dropped below 2.5 ng/mL; otherwise, the rHuEPO dose is reduced by 25%. ERFE was measured within 1 day after the first and fourth injections of rHuEPO or saline. Erythropoietic responses (HGB and RBC) were followed up for 1 month after the initial rHuEPO dose. The rHuEPO dose titration in Week 2 was based on the change of HGB (Δ*H*GB) in “Week 1” or the change of ERFE (ΔERFE) after the first injection of rHuEPO. The rHuEPO dose titration in Week 3 was based on the change of HGB (ΔHGB) in “Week 2” or the change of ERFE (Δ*E*RFE) after the fourth injection of rHuEPO. The increase in ERFE at 4 h post-rHuEPO of 2.5 ng/mL was determined based on the regression equation of ERFE and HGB in CKD rats (Figure 4A), where an increase in HGB of 2 g/dL corresponds to an increase in ERFE of 2.5 ng/mL. Blood samples (100–150 μL) were collected weekly via tail vein and reevaluated for both groups. The percentage of rHuEPO dose consumption was calculated by dividing the total rHuEPO dose consumed by the initiating dose.

### Hematologic Parameters and ERFE Assay

Hemoglobin (HGB, g/dL) and red blood cell (RBC, 10^12^ cells/L) were assayed on a BC2800VET Hematology Analyzer (Mindray). ERFE measurement was determined using a validated enzyme-linked immunosorbent assay (ELISA) kit (ER1573, FineTest, Wuhan, China). All procedures were performed following the manufacturer’s instructions. To mitigate the influence of baseline ERFE, the relative change of ERFE (%) was calculated by dividing the change of ERFE from baseline by the baseline level and multiplying by 100.

### Flow Cytometry Analysis

To systematically determine the depletion and recovery of erythroid precursors after carboplatin treatment, FACS analysis was performed consecutively for 2 weeks after carboplatin injection. Flow cytometry was performed on bone marrow and spleen. The gating strategy is shown in Figure 3B. Specifically, this protocol allows for the identification and isolation of three populations from the bone marrow and spleen: P1, pro-, basophilic, polychromatophilic erythroblasts; P2, orthochromatic erythroblasts; P3, reticulocytes and RBCs.^29^ Bone marrow was harvested by flushing femurs with PBS with 0.5% BSA. The spleen was dissociated into single-cell suspensions in PBS with 0.5% BSA. The absolute cell counts of marrow and spleen cells were determined by using counting beads (Invitrogen). Cells were pretreated with rat antibody IgG (BD Biosciences) for 30 min to block unspecific immunoglobulin binding and subsequently stained with PE-conjugated antibody to rat CD71 (BD Biosciences) and HIS49 rat antibody (BD Biosciences) and incubated on ice for 30 min in the dark. Cells were then stained with the APC rat antibody (BD Biosciences). Flow cytometry was performed on a BD LSR Fortessa Cell Analyzer (BD Biosciences).

### PK/PD Modeling

**Figure 1:**
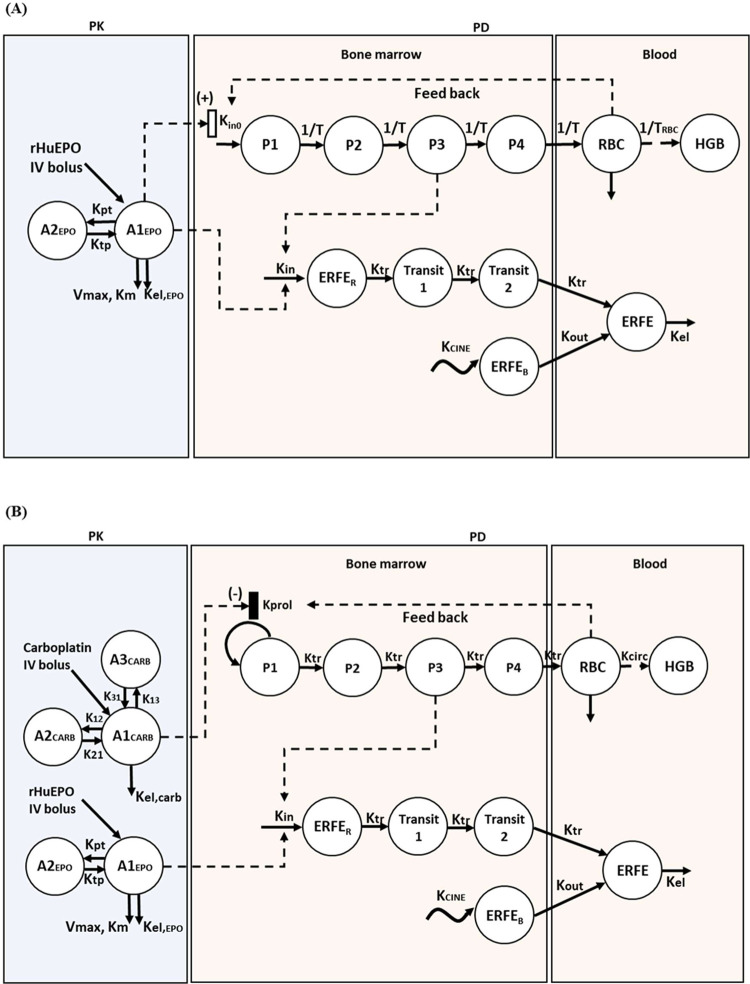
Model structure for CKD rats (A) and CIA rats (B).

A PK/PD model was developed to characterize the kinetic profiles of ERFE, HGB, and RBC after rHuEPO treatment and dissect the impact of key factors on ERFE induction by rHuEPO in CIA rats and CKD rats. The PK/PD model structure for CKD rats (Figure 1A) and CIA rats (Figure 1B) was shown.

#### PK Model

The PK model of rHuEPO was described by a two-compartment model with linear and nonlinear eliminations. The PK of carboplatin was described by a three-compartment model with a linear elimination. PK parameters for rHuEPO and carboplatin (Table S1) were fixed based on previous studies conducted in CKD rats or CIA rats assuming the same PK for rHuEPO and carboplatin in rats.^28,30^ The differential equations of the rHuEPO PK model are as follows
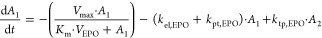
1
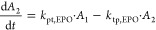
2where *A*_1_ and *A*_2_ are the amounts of rHuEPO in the central and peripheral compartments, respectively. *A*_1_(0) = DOSE_rHuEPO_iv_, *A*_2_(0) = 0. *V*_max_ and *K*_m_ are the parameters associated with nonlinear elimination of rHuEPO; *k*_el,EPO_ is the first-order rate for linear elimination of rHuEPO. *k*_pt,EPO_ and *k*_tp,EPO_ are the intercompartmental rate constants. DOSE_rHuEPO_iv_ is the dose of rHuEPO administered through i.v. injection.

The differential equations of the carboplatin PK model are as follows
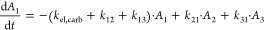
3
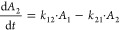
4
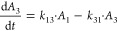
5where *A*_1_ is the amount of rHuEPO in the central compartment; *A*_2_ and *A*_3_ are the amounts of rHuEPO in the peripheral compartments. *A*_1_(0) = DOSE_CAR_iv_, *A*_2_(0) = 0, and *A*_3_(0) = 0. *k*_el,carb_ refer to the first-order elimination rate for carboplatin. *k*_12_, *k*_21_, *k*_13_, and *k*_31_ are the intercompartmental rate constants. DOSE_CAR_iv_ is the dose of carboplatin administered through i.v. injection.

The PD model consists of two submodels, including indirect response models describing proliferation and maturation of erythroid precursor cells^31^ and the ERFE model.

#### Erythroid Response Model

The delayed maturation from erythroid precursors to red blood cells was characterized by indirect response models, where cell population compartments represent burst-forming unit erythroid cells (BFU-Es), colony-forming unit erythroid cells (CFU-Es), erythroblasts (EBs) at different stages (i.e., Pro-, Baso-, Poly-, and Ortho-EBs; represented by P3), reticulocyte (RETs), and red blood cell (RBC).^28,32,33^ The proliferation (*K*_prol_) of the precursor cell was either increased with rHuEPO stimulation or decreased when exposed to carboplatin. Additionally, red blood cells (RBCs) have a feedback effect on precursor cell proliferation.^34,35^

The differential equations of the CKD PD model describing the proliferation and maturation of erythroid precursor cells are as follows^28,32,33^
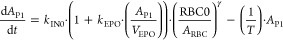
6
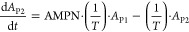
7
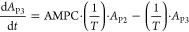
8
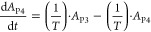
9
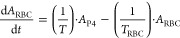
10
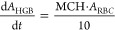
11where *A*_P1_, *A*_P2_, *A*_P3_, *A*_P4_, *A*_RBC_, and *A*_HGB_ are BFU-Es CFU-Es, EBs (i.e., Pro-, Baso-, Poly-, and Ortho-EBs), RETs, red blood cells, and hemoglobin. Under initial conditions, , , *A*_*P*3_(0) = RET0, , . AMPN and AMPC are the scaling factors that represent the number of CFU-E cells produced by one BFU-E cell and the number of erythroblasts produced by one CFU-E cell, respectively; AMPN = AMPC = 2^5^. *RBC*0 is the initial voltage for red blood cell. *T* and *T*_RBC_ are the average lifespans of precursor cells and red blood cells, respectively. *K*_IN0_ is the cell proliferation rate constant, which is defined as . It is affected by a feedback mechanism and rHuEPO stimulation . The feedback loop accounts for the negative effect of circulating RBC cells on cell proliferation, which is essential to capture the rebound of cells. rHuEPO stimulation is incorporated as , where  is the rHuEPO concentration and *k*_EPO_ is the first-order stimulatory effect of rHuEPO.

The differential equations of the CIA PD model describing the proliferation and maturation of erythroid precursor cells are as follows^34,35^
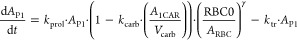
12
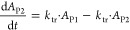
13
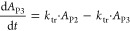
14
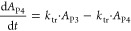
15
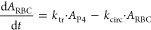
16
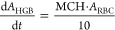
17where *A*_P1_, *A*_P2_, *A*_P3_, and *A*_P4_ refer to BFU-Es, CFU-Es, EBs, and RETs, respectively. The initial conditions for *A*_P1_, *A*_P2_,*A*_P3_,*A*_P4_,*A*_RBC_ are defined as RBC0. The proliferation of erythroid progenitor cells was described by a proliferation rate constant (*k*_prol_). The self-renewal of cells was dependent on the number of existing cells (*A*_P1_), the feedback effect of circulating RBCs , and impaired by carboplatin-induced myelosuppression . To minimize the number of parameters to be estimated, the time delay of cell maturation was described by three transit compartments (*k*_tr_) and it is assumed that *k*_prol_ = *k*_circ_ = *k*_tr_ = (*N* + 1)/MTT, where *N* is the number of transit compartments.

#### ERFE Model

To characterize the double peak kinetics of ERFE, the ERFE model assumes a simultaneous input via two pathways.^36^ Under baseline conditions (i.e., independent of the rHuEPO stimulation), ERFE exhibited a circadian rhythm, which was characterized by a time-dependent input rate (*K*_CINE_) and a first-order elimination rate (*K*_out_).^37^ Upon stimulation by rHuEPO, ERFE is secreted from erythroblasts in the bone marrow and released into the circulation. The delay in the release of ERFE is characterized by two transit compartments.^38^ The induction of ERFE is assumed to be proportional to both rHuEPO stimulation and precursor cell mass. The intrinsic potency of ERFE’s response to rHuEPO was characterized by EC_50_, which equals the rHuEPO concentration required to induce 50% of the maximum ERFE induction. The differential equations of the ERFE PD model are as follows
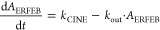
18where *A*_ERFEB_ represents ERFE under baseline conditions: . A time-dependent production rate (*k*_CINE_) was used to describe the circadian production of ERFE.^24,39^*k*_CINE_ is defined as . *k*_out_ is the first-order elimination rate of circadian ERFE, and cos and sin represent the cosine and sine functions in trigonometry, respectively
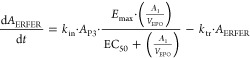
19
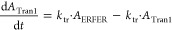
20
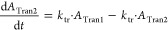
21
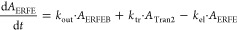
22where *A*_ERFER_ represents the ERFE induction by rHuEPO, *A*_Tran1_ and *A*_Tran2_ represent the two transit compartments for ERFE, and *A*_ERFE_ is the circulating ERFE (including baseline ERFE and ERFE induction by rHuEPO). The initial conditions for *A*_ERFER_, *A*_Tran1_, and *A*_Tran2_ are zero, and *A*_ERFE_(0) = *A*_ERFEB_(0). The ERFE induction by rHuEPO is assumed to be proportional to erythroblasts (*A*_P3_) and rHuEPO stimulation . And the delay in the release of ERFE from precursors to circulation upon rHuEPO stimulation was described using two transit compartments (*k*_tr_). To minimize the number of parameters to be estimated, the production rate of ERFE (*k*_in_) and the elimination rate of ERFE (*k*_el_) are assumed to be equal to those of *k*_tr_.

Interindividual variability (IIV) was fixed as zero. The residual variance was described by a proportional error model *Y* = *Ŷ*(1 + ε), where *Y* denotes the observed concentration, *Ŷ* is the predicted individual concentration, and ε is the random residual error, which follows a normal distribution with a mean of 0 and a variance of σ^2^.

### Model-Based Simulation

To investigate the impact of key factors on ERFE kinetics, model-based simulations were performed. First, the parameters were fixed to the estimated parameters for CKD rats and CIA rats. Second, CKD rats having distinct rHuEPO responsiveness levels were considered, with EC_50_ corresponding to high responsiveness (EC_50_ = 10), medium responsiveness (EC_50_ = 100), and low responsiveness (EC_50_ = 1360). CIA rats with varying precursor cell masses were considered: A(P3) times 1 (representing low precursor cell mass), A(P3) times 2 (representing medium precursor cell mass), and A(P3) times 3 (representing high precursor cell mass). Lastly, ERFE-time profiles were simulated based on two rat models at three dose levels (0, 450, and 1350 IU/kg). Additionally, to evaluate the myelosuppressive effect of carboplatin on the precursor cell responsive to ERFE production, the time course of the P3 cell population was simulated.

### Data Analysis

#### Parameter Estimation and Model Evaluation

PK/PD model fittings were performed by nonlinear regression using NONMEM 7.5 (Version 7.4.0; ICON Development Solutions). The model evaluation encompassed both the drop in the objective function value (OFV) and the assessment of goodness-of-fit. A reduction of 3.84 in the OFV was statistically significant at a 95% confidence interval. Diagnostic plots were employed to evaluate goodness-of-fit, including comparisons between population-predicted concentration and observed concentration, individual-predicted concentration and observed concentration, conditional weighted residual (CWRES) versus population-predicted concentration, and CWRES versus time. Additionally, the final model’s performance was assessed using a prediction-corrected visual predictive check (pcVPC). The 95% confidence intervals (CIs) for observed versus predicted concentration–time profiles were visually compared.

#### Correlation Analysis

Linear regression was performed to test the association between the maximum induction of ERFE and HGB responses in CKD and CIA rats following rHuEPO treatment. A previous study conducted in healthy rats demonstrated that the initial peak of ERFE represented the immediate induction of ERFE by rHuEPO, whereas the second peak of ERFE resulted from the circadian rhythm of endogenous EPO.^24^ To adjust for the baseline difference, the change of ERFE at 4 h post-rHuEPO was selected as a predictor representing ERFE maximum release in response to rHuEPO injection. And giving HGB response occurs only after at least 1 week in rats; the change of HGB within 1 week was selected as the end point.

#### ROC Curve Analysis

Receiver operating characteristic (ROC) curve analysis was performed to test the diagnostic power of ERFE in predicting rHuEPO hyporesponsiveness in CIA rats. Specifically, hyporesponsiveness to rHuEPO was defined as an increase in HGB of less than 1g/dL from baseline within 1 week. The changes in ERFE at 4 h post and 10 h post-rHuEPO injections were evaluated as predictors by the ROC curve. The optimal ERFE cutoff value for predicting rHuEPO hyporesponsiveness was estimated based on the Youden index (J-index), which ranges from 0 (no diagnostic value) to 1 (perfect test).

Statistical significance was assessed by Student *t* test or one-way ANOVA with the Tukey test. Analysis and plotting were performed using R Software (version 3.5.2) via Rstudio (Version 1.1.456).

## Results

### Short-Term ERFE and Long-Term Hematological Responses to rHuEPO in CKD Rats and CIA Rats

Two anemic rat models were established to determine the ERFE and erythroid responses to rHuEPO under conditions of impaired EPO responsiveness (i.e., CKD-associated anemia) and reduced precursor cells (i.e., CIA). The adenine-treated rats developed anemia, evidenced by a reduction of more than 2 g/dL in HGB compared to healthy controls, and the anemia persisted for at least 1 month (Figure S3A,B). The carboplatin-treated group exhibited a rapid decline in RBC and HGB levels, reaching a nadir of 8 g/dL on day 14. Both HGB and RBC levels in CIA rats gradually rebounded to normal after 1 month (Figure S3C,D).

**Figure 2:**
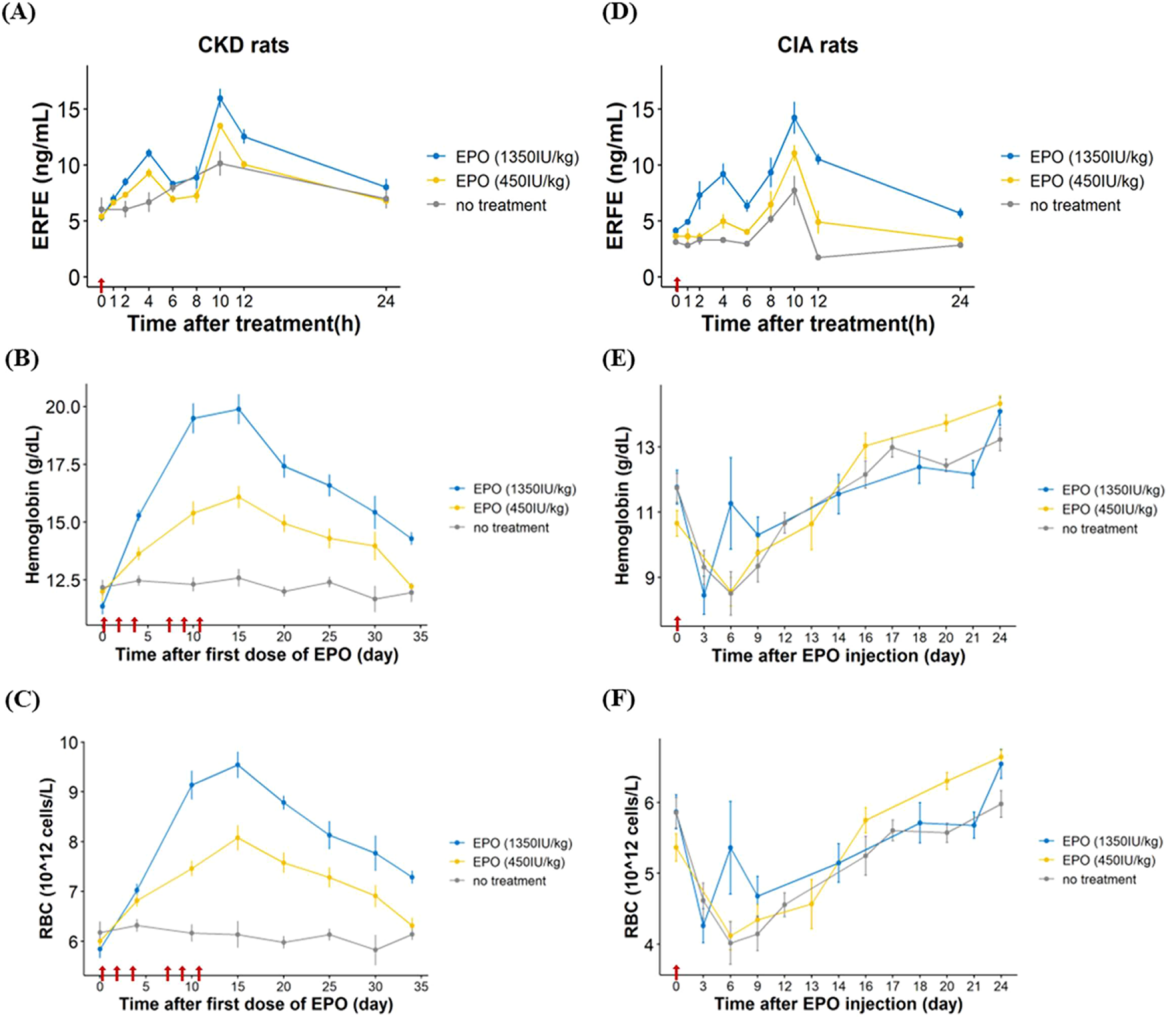
ERFE kinetics after rHuEPO treatment (1350 or 450 IU/kg) or saline treatment in CKD rats (*n* = 5 for no treatment group, *n* = 6 for 450 IU/kg EPO treatment group, *n* = 6 for 450 IU/kg EPO treatment group, (A–C)) and CIA rats (*n* = 9 for each group, (D–F)). The data points represent the mean, and the error bars indicate the standard deviation. The arrow represents the rHuEPO administration.

We then determined the kinetic profiles of plasma ERFE (Figure 2A) and hematological responses (Figure 2B,C) after repeated rHuEPO dosing in CKD rats. The ERFE increased and peaked at 4 h and 10 h after the first dose of rHuEPO at either 450 or 1350 IU/kg and returned to baseline by 24 h. In the nontreatment group, CKD rats exhibited a single peak of ERFE at 10 h after saline injection. For long-term HGB and RBC responses after repeated doses of rHuEPO for 2 weeks, CKD rats exhibited a rapid increase in mean HGB and RBC in a dose-dependent manner and peaked at the end of treatment.

In CIA rats, to replicate the kinetic profiles of ERFE and hematological parameters in hyporesponders after rHuEPO treatment, only a single dose of rHuEPO was given. The ERFE kinetics in CIA rats (Figure 2D) were similar to those in CKD rats. Notably, the ERFE peak at 4 h post-rHuEPO was only apparent in high-dose (1350 IU/kg) group rather than low-dose group (450 IU/kg) in CIA rats. The hematological response to rHuEPO (Figure 2E,F) also exhibited high interindividual variability, with some CIA rats showing hyporesponsiveness to rHuEPO.

### Increased Baseline ERFE Protein Levels But Decreased ERFE Induction by rHuEPO in Anemic Rats Compared to Those in Healthy Rats

**Figure 3:**
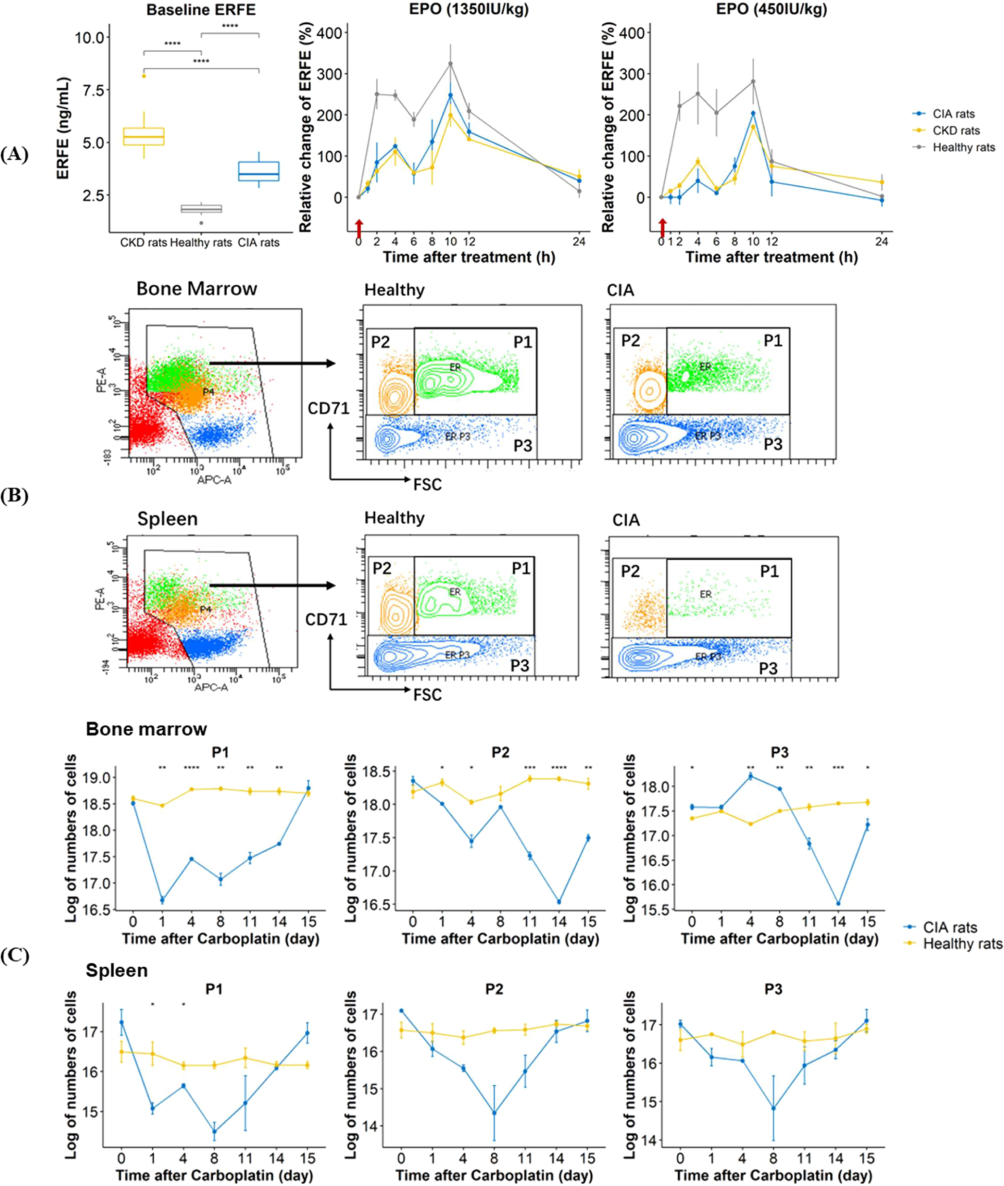
Baseline ERFE levels and ERFE kinetics after rHuEPO treatment at 1350 IU/kg or 450 IU/kg in CKD rats, CIA rats and healthy rats (*n* = 7 for no treatment group, *n* = 9 for 450 IU/kg EPO treatment group, *n* = 9 for 1350 IU/kg EPO treatment group). The arrow represents rHuEPO administration. (A). Flow cytometry gating strategy (B), P1, P2, and P3 numbers in the bone marrow and spleen in CIA rats (*n* = 3 per day) during the development of disease versus healthy rats (*n* = 3 per day). P1, pro-, basophilic, polychromatophilic erythroblasts; P2, orthochromatic erythroblasts; P3, reticulocytes and RBCs. (C). The data are presented as mean ± standard deviation. *P < 0.05; **P < 0.01; ***P < 0.001; ****P < 0.0001 using one-way ANOVA followed by Tukey test (A) and Student *t* test (C).

We then compared the baseline ERFE and the relative change in ERFE after rHuEPO treatment in anemic rats versus healthy rats. The baseline ERFE protein levels in CKD rats and CIA rats were significantly higher than that in healthy rats (*P* ≤ 0.0001) (Figure 3A). Compared with healthy rats, both CKD and CIA rats exhibited a lower relative change in ERFE levels at 4 h after rHuEPO treatment (1350 or 450 IU/kg), indicating an impaired ERFE response to rHuEPO (Figure 3A).

As ERFE was mainly secreted by erythroid precursor cells, we hypothesized that the decreased ERFE induction in CIA rats is primarily due to a decrease in precursor cells responding to rHuEPO. We further investigated the kinetics of precursor cell populations by flow cytometry analysis in CIA rats as compared with those in healthy rats (Figure 3C). The kinetics of precursors included a delayed decline, nadir, recovery, overshoot, and return to the baseline. We observed that carboplatin depleted almost all marrow erythroid precursors with less effect observed on splenic erythroid precursor cells. This is consistent with the myelosuppression effect of carboplatin on the bone marrow.^40^

### Short-Term ERFE Response Predicts Long-Term HGB Response and rHuEPO Hyporesponsiveness in Anemic Rats

**Figure 4:**
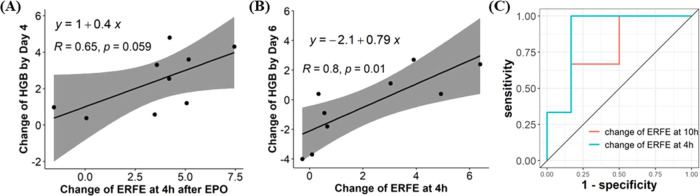
Correlation analysis of HGB response and the change of ERFE after rHuEPO injection in CKD rats (*n* = 9, A) and CIA rats (*n* = 9, B). Regression equations, correlation coefficients, and P-values are reported in each panel. The ROC curve analysis for the change of ERFE at 4 h and the change of ERFE at 10 h conducted in CIA rats (*n* = 9, C).

Based on the observations of ERFE and HGB response to rHuEPO and the physiological function of ERFE, we asked whether ERFE could predict the HGB response after rHuEPO treatment in anemic rats. Correlation analysis of the long-term HGB response and the change of ERFE after rHuEPO injection was conducted on CKD rats (Figure 4A) and CIA rats (Figure 4B). We observed a positive correlation between the change of ERFE at 4 h post-rHuEPO and the change of HGB by day 4 after rHuEPO treatment in CKD rats (*R* = 0.65, *P* = 0.059). In CIA rats, the change of ERFE at 4 h post-rHuEPO was significantly correlated with the change of HGB by day 6 after rHuEPO treatment (*R* = 0.8, *P* = 0.01).

In addition, being aware of the high variability of the HGB response in CIA rats, we investigated if ERFE could predict rHuEPO hyporesponsiveness. At ROC analysis, both the change of ERFE at 4 h post-rHuEPO from baseline (AUC = 0.89) and the change of ERFE at 10 h post-rHuEPO from baseline (AUC = 0.78) were predictors of rHuEPO hyporesponsiveness (Figure 4C). The optimal ERFE cutoff value for predicting rHuEPO hyporesponsiveness in CIA rats was estimated to be 1.85 ng/mL.

### ERFE-Based Dose Titration Leads to a rHuEPO-Sparing Effect

**Figure 5:**
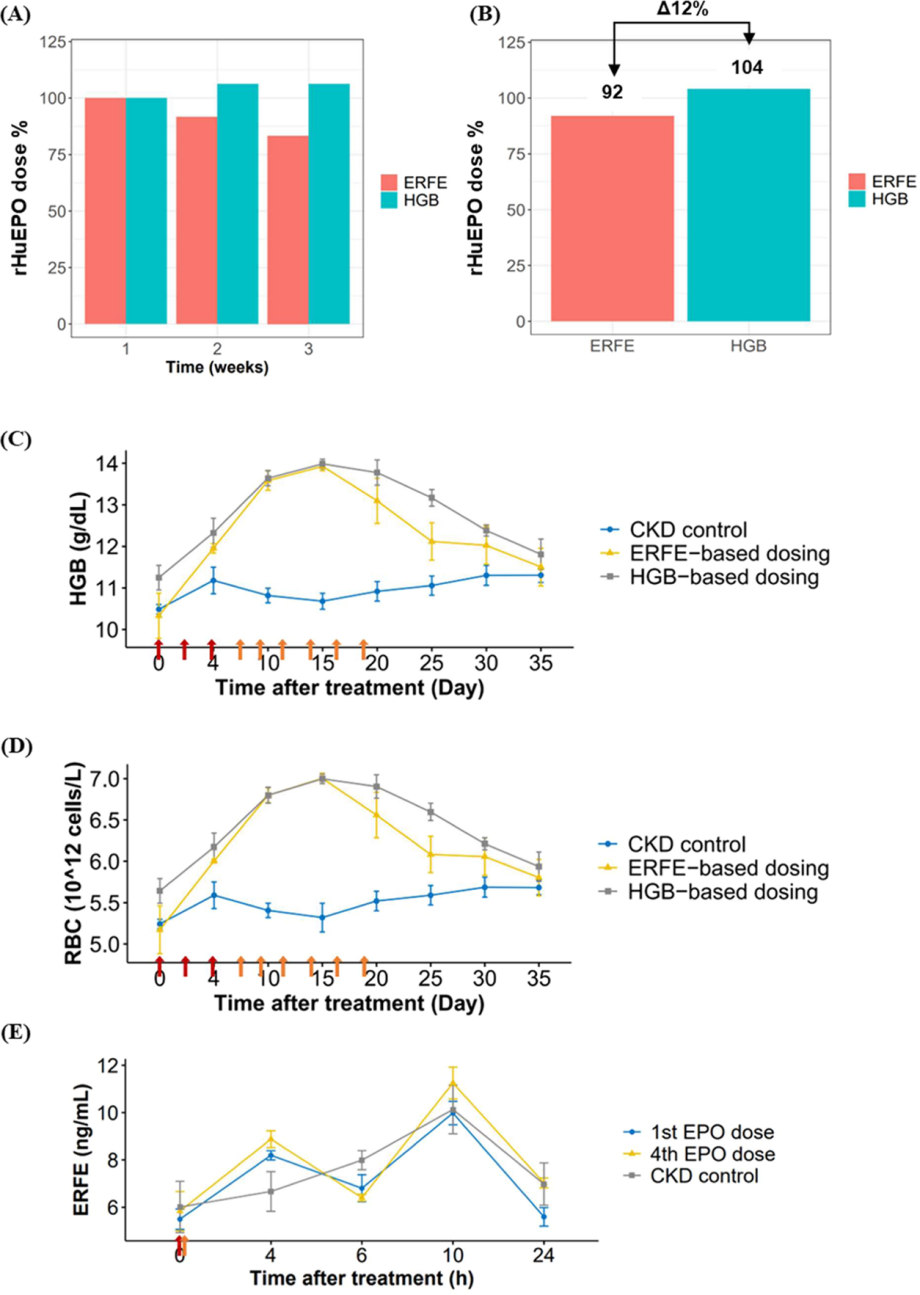
Percentage of rHuEPO consumed following ERFE-based dosing or HGB-based dosing in week 1, week 2, and week 3 (A), the total rHuEPO consumption and reduction of rHuEPO dose (in %) following ERFE-based dosing or HGB-based dosing in CKD rats (B). The time course of HGB and RBC after rHuEPO treatment following ERFE-based dosing (*n* = 3) or HGB-based dosing (*n* = 4) or no treatment (*n* = 3) in CKD rats (C, D). ERFE kinetics after rHuEPO treatment at the 1st dose or 4th dose or no treatment in CKD rats (E). The red arrow represents rHuEPO initial dose; the orange arrow represents rHuEPO maintenance dose.

Given that higher rHuEPO dose is associated with increased mortality in patients with CKD,^41^ we investigated if using ERFE-based rHuEPO dose titration could reduce the rHuEPO dose required to correct anemia in CKD rats. We conducted a separate study on CKD rats to compare the effect of dose titration methodology on the rHuEPO consumption, hematological parameters, and ERFE response. After a 3-week rHuEPO treatment following the dose titration rule, CKD rats following ERFE-based dose titration consumed less weekly rHuEPO dose as compared to the HGB-based dosing group during the maintenance period (Figure 5A). Compared to HGB-based dosing, ERFE-based dosing in total resulted in a 12% reduction of the cumulative rHuEPO dose (Figure 5B) throughout the treatment period. Both treatment groups achieved target HGB levels in CKD rats; however, the HGB-based dosing group exhibited a longer duration of overshooting of HGB above 13 g/dL by day 25 (Figure 5C).

### Mechanistic PK/PD Model Revealed the Impact of Key Factors Involved in ERFE Induction by rHuEPO

**Figure 6:**
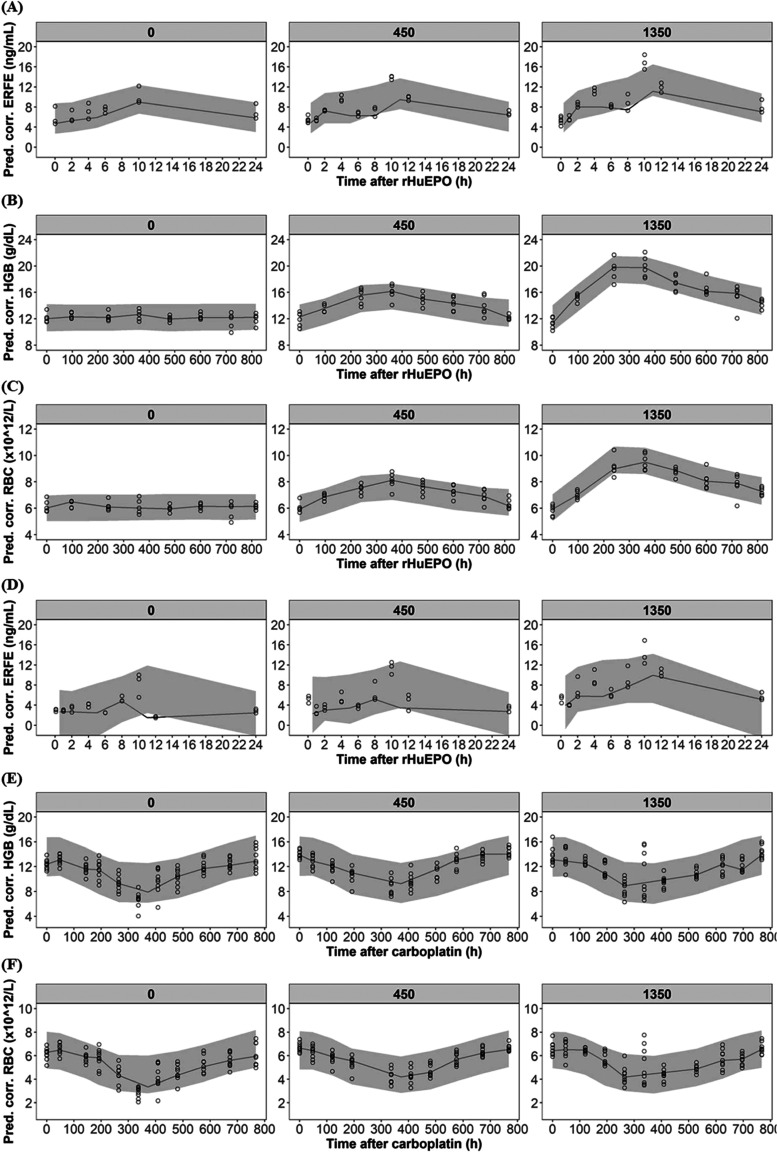
PcVPC for ERFE, HGB, and RBC in CKD rats (A–C) and CIA rats (D–F).

Next, we established a mechanistic PK/PD model to describe the ERFE and HGB responses to rHuEPO in CKD and CIA rats (Figure 1). The transit model adequately described the delayed release of ERFE that peaked around 4 h after rHuEPO stimulation and significantly increased the goodness-of-fitting of the model. The pcVPC results showed that the 50th percentile from the observations fell within the 95% confidence interval derived from model predictions for ERFE, HGB, and RBC in CKD rats (Figure 6A–C) and CIA rats (Figure 6D–F). The goodness-of-fit plots for the CKD rat model (Figure S4) and CIA rat model (Figure S5) indicated that the model predictions agreed well with the observations.

**Figure 7:**
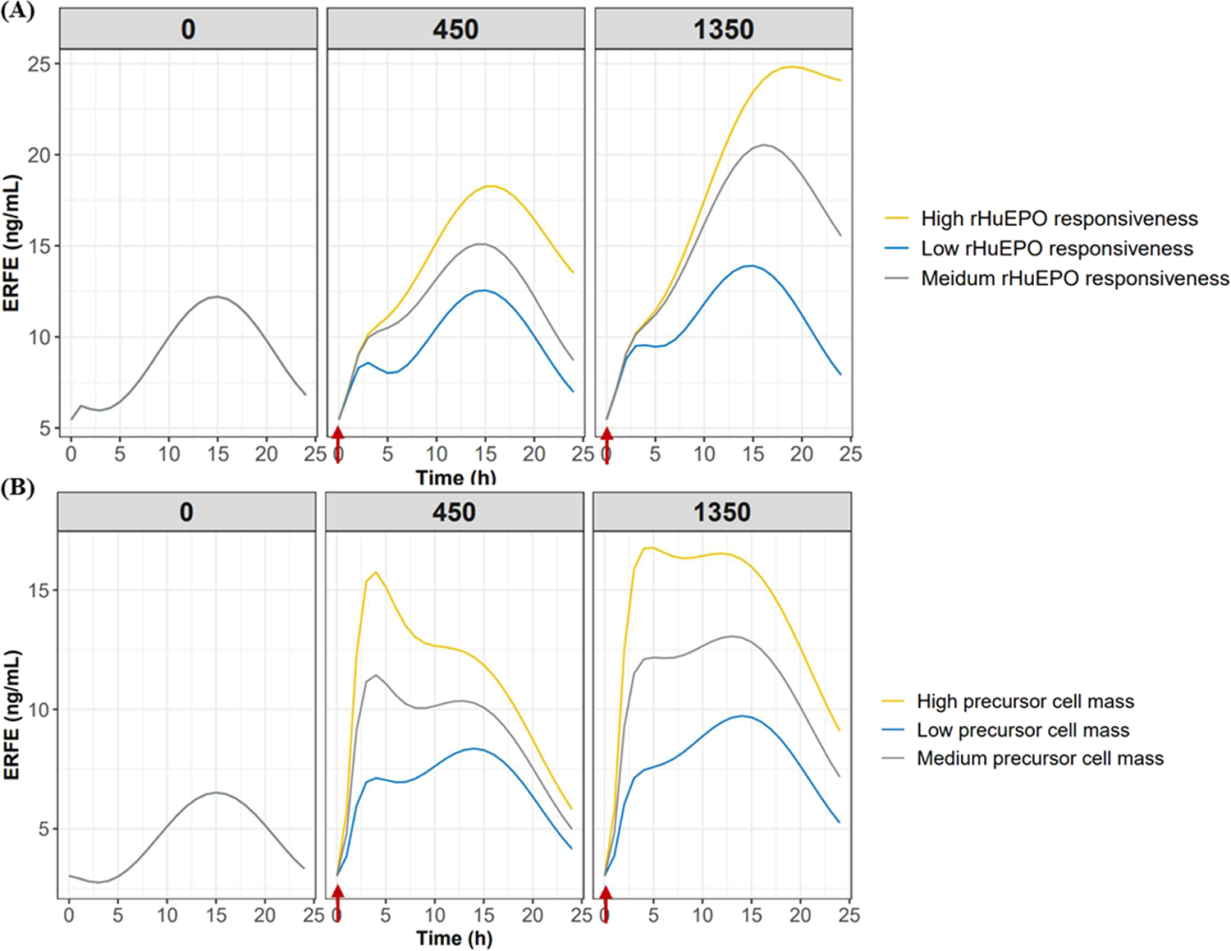
Simulated time course of ERFE in CKD rats with distinct levels of EPO responsiveness (from low to high) (A). The simulated ERFE kinetics in CIA rats with varying precursor cell mass levels (from low to high) (B). The simulated time course of P3 representing the major cell population producing ERFE in CIA rats. The arrow represents rHuEPO administration (C).

PD parameters for CKD rats and CIA rats are listed in Tables 1 and2. A higher estimated EC_50_ in CKD rats (1360 mIU/mL, RSE 69.9%) than in CIA rats (126 mIU/mL, RSE 31.2%) indicated that the impaired EPO responsiveness predominantly contributes to the reduced induction of ERFE following rHuEPO administration in CKD rats. In contrast, a decline in precursor cells (P3) that reached a nadir by the time of rHuEPO administration (Figure S6) may lead to a blunted ERFE response to rHuEPO in CIA rats. The simulated time course of ERFE showed that impaired rHuEPO responsiveness (Figure 7A) and reduced precursor cell mass (Figure 7B) lead to blunted ERFE dynamics at different rHuEPO levels.

**Table 1:** Parameter Estimates of the CKD-Associated Anemia Rat PD Model^a^

parameter (unit)	description	estimate (RSE%)
*T*_RBC_ (h)	mean lifespan for red blood cells	1280 (13.2)
*T* (h)	mean lifespan for precursor cells	20.7 (8.5)
RBC0	mean baseline of RBC counts	6.15 (0.9)
MCH (pg·cell^–1^)	mean corpuscular hemoglobin	20.2 (0.9)
γ	feedback factor of RBC	3.9 (19.9)
*K*_EPO_	stimulatory effect of EPO on precursor cells	0.0106 (19.2)
*K*_out_ (h^–1^)	first-order elimination constant of circadian ERFE	2.82 (18.8)
RM (ng·mL^–1^)	mean baseline mesor for ERFE circadian rhythm	7.25 (8.6)
RA (ng·mL^–1^)	amplitude for ERFE circadian rhythm	2.51 (15.9)
*t*_peak_ (h)	peak time for ERFE circadian rhythm	14.9 (4)
*E*_max_ (cell^–1^)	maximum ERFE induction by rHuEPO per precursor cell	47.2 (37.5)
EC_50_ (mIU·mL^–1^)	the rHuEPO concentration required to induce 50% of the maximum ERFE induction	1360 (69.9)
*K*_tr_ (h^–1^)	transit rate constant for ERFE	2.25 (20.5)
σ_RBC_ (%)	proportional error for RBC	0.495 (6.2)
σ_HGB_ (%)	proportional error for HGB	1.03 (6.2)
σ_ERFE_ (%)	proportional error for ERFE	1.54 (8.1)

a HGB, hemoglobin; RBC, red blood cell; ERFE, erythroferrone; RSE, relative standard error. Residual errors are expressed as coefficients of variation (%) with RSE on the approximate standard deviation scale (standard error/variance estimate)/2.

**Table 2:** Parameter Estimates of the CIA Rat PD Model^a^

parameter (unit)	description	estimate (RSE%)
*K*_carb_ (h^–1^)	killing effect of carboplatin on the precursor cell	0.172 (4.6)
MTT (h)	mean transit time for the maturation of the precursor cell	200 (3.1)
RBC0	mean baseline of RBC counts	6.46 (1.1)
MCH (pg·cell^–1^)	mean corpuscular hemoglobin	21.1 (1.1)
γ	feedback factor of RBC	0.189 (8)
*K*_out_ (h^–1^)	first-order elimination constant of circadian ERFE	1.16 (19.9)
RM (ng·mL^–1^)	mean baseline mesor for ERFE circadian rhythm	9.03 (18.2)
RA (ng·mL^–1^)	amplitude for ERFE circadian rhythm	3.69 (16.5)
*t*_peak_ (h)	peak time for ERFE circadian rhythm	15.1 (7.4)
*E*_max_ (cell^–1^)	maximum ERFE induction by rHuEPO per precursor cell	1.16 (14.5)
EC_50_ (mIU·mL^–1^)	the rHuEPO concentration required to induce 50% of the maximum ERFE induction	126 (31.2)
*K*_tr_ (h^–1^)	transit rate constant for ERFE	2.26 (14.9)
σ_RBC_ (%)	proportional error for RBC	0.748 (4.2)
σ_HGB_ (%)	proportional error for HGB	1.51 (4.2)
σ_ERFE_ (%)	proportional error for ERFE	2.2 (7.9)

a HGB, hemoglobin; RBC, red blood cell; ERFE, erythroferrone; RSE, relative standard error. Residual errors are expressed as coefficients of variation (%) with RSE on the approximate standard deviation scale (standard error/variance estimate)/2.

## Discussion

Understanding the kinetics of ERFE in pathological states and its influencing factors can provide valuable insights into its utility as a biomarker. In this study, utilizing CKD and CIA rat models combined with a modeling-based approach, we elucidated the dynamics of ERFE in response to rHuEPO and the impact of precursor cell mass and rHuEPO responsiveness. This study validated the utility of ERFE in predicting the HGB response and rHuEPO hyporesponsiveness. Furthermore, we provided compelling evidence that ERFE-based dose titration reduced the rHuEPO dosage and minimized the risk of HGB overshooting in CKD rats.

ERFE expression has been upregulated in murine models with inefficient erythropoiesis, such as anemia of inflammation^42^ and in patients with myelodysplastic syndromes (MDS),^43^ β-thalassemia,^26^ and malarial anemic.^27^ Indeed, both CIA rats and CKD rats exhibited elevated ERFE protein levels under baseline conditions compared with healthy rats (Figure 3A). The baseline ERFE is expected to exhibit a circadian rhythm because it is a downstream responder of endogenous EPO, which follows a circadian rhythm.^44^ The circadian kinetics of baseline ERFE was described by a time-dependent input function.^24^ The higher baseline ERFE in anemic rats could be attributed to increased ERFE production, which occurs as a secondary effect of increased endogenous EPO levels in response to anemia. Several studies have reported a transient increase of endogenous EPO levels after receiving chemotherapy in patients^45,46^ and in rats^35^ and the upregulated EPO level is correlated with the decrease in erythroid precursor cell mass.^47^ Additionally, inflammatory factors other than anemia may also contribute to the increase of baseline ERFE levels.^48^

We further investigated the factors governing ERFE induction by rHuEPO other than baseline ERFE. Nevertheless, assessing the impact on ERFE induction by rHuEPO is challenging due to the complex interplay of pathological factors and the influence of its circadian baseline. Hence, we employed a mechanism-based PK/PD model to quantitatively assess the pathological impact on ERFE dynamics and supported this mechanism through experimentation. In healthy rats, repeated rHuEPO dosing could enhance ERFE response and expand erythroblasts.^21^ This suggested a positive correlation between ERFE and erythroid precursor mass. ERFE, as a downstream effector of EPO-EPOR signaling, could also be affected by the EPO responsiveness. We thus incorporated these two factors in the PK/PD model by assuming that ERFE induction by rHuEPO is proportional to the precursor cell mass (P3) and the rHuEPO stimulatory effect. This model is frequently used in the literature to describe the dynamics of tumor cell biomarkers^49^ and the signal transduction process.^38^ The mechanistic model captured the ERFE kinetics well in CKD rats and CIA rats. The simulated ERFE kinetics of anemic rats (Figure 7) indicate that the diminished precursor cell mass and/or impaired rHuEPO responsiveness leads to reduced induction of ERFE by rHuEPO. As supported by experimental data (Figure 3), CIA rats exhibited a large depletion of marrow erythroid cells across different cell populations, particularly reaching a nadir for the precursor cell that primarily produce ERFE by the time of rHuEPO treatment. Given that the bone marrow is the major organ for stress erythropoiesis in humans and rats,^50^ the deduction of precursor cell mass could result in impaired ERFE induction by rHuEPO in CIA rats. In CKD rats, we postulate that impaired EPO responsiveness (as suggested by a higher EC_50_) predominantly contributes to the blunted ERFE response to rHuEPO. The impaired EPO responsiveness in CKD rats could be attributed to excess proinflammatory cytokines.^51^ These cytokines may directly downregulate EPOR expression or indirectly inhibit the growth of erythroid progenitor cells that express EPOR at very high levels^52^ and consequently leads to the decline of downstream ERFE responses. These findings suggest the potential of ERFE as a biomarker for predicting rHuEPO efficacy, as reduced precursor cell mass or impaired rHuEPO responsiveness is expected to lead to compromised erythroid response.

Indeed, anemic rats of CKD and CIA exhibited a lower induction of ERFE at 4 h post-rHuEPO, which is associated with restricted HGB improvement in comparison to healthy rats following rHuEPO treatment at the same levels.^24^ We also showed that the maximum induction of ERFE at 4 h post-rHuEPO could predict the long-term HGB response in anemic rats of CKD and CIA. Previous studies reported that ERFE increases in response to rHuEPO in mice and humans^17,23^ and its peak value could serve as a surrogate to predict peak HGB in healthy rats.^24^ However, peak ERFE concentration has a limited value in reflecting the ERFE response to rHuEPO in anemic rats. This is because the peak ERFE is also influenced by baseline ERFE, which has been reported to negatively correlate with baseline levels of HGB in anemic patients.^25,26^ For instance, a hyporesponder with a lower than average HGB levels (which is associated with higher than average baseline ERFE) and decreased ERFE induction by rHuEPO could have a higher peak ERFE, thus blurring the results of estimation. Therefore, the peak ERFE does not provide an accurate estimation of the ERFE released in response to rHuEPO treatment in anemic patients. Given that the second peak of ERFE mainly reflects the ERFE induced by endogenous EPO,^24^ the initial change of ERFE at 4 h post-rHuEPO was used as a surrogate to reflect the maximum response of ERFE to rHuEPO in anemic rats. The positive correlation of the ERFE maximum induction and the HGB response was in line with the physiological function of ERFE in stress erythropoiesis.^42^

Importantly, we demonstrated that ERFE at 4 h post-treatment well predicts rHuEPO hyporesponsiveness in CIA rats. As expected, CIA rats exhibited large interindividual variability in HGB response after rHuEPO treatment at both dose levels. The ROC curve analysis revealed that the change in ERFE at 4 h is a better predictor of rHuEPO hyporesponsiveness compared to the change in ERFE at 10 h post-treatment. This aligns with the physiological interpretation of ERFE kinetics, where the initial peak is driven by rHuEPO, and the second peak is primarily dominated by the circadian rhythm of endogenous EPO.^21^ Based on the ROC curve, the estimated optimal ERFE cutoff value for predicting rHuEPO hyporesponsiveness was 1.85 ng/mL in CIA rats. Early prediction of rHuEPO hyporesponsiveness would allow switching to other treatments and may circumvent the resulting adverse effects in patients. This is of clinical importance because around 50% of CIA patients are resistant to rHuEPO.^53^

Moreover, our study revealed that ERFE-based dosing reduced the required rHuEPO dose by up to 12% compared to HGB-based dosing in CKD rats while achieving the target HGB level. Considering the longer dosing period in clinical settings, ERFE-based titration may have an even greater impact on reducing the rHuEPO dose among patients. This rHuEPO-sparing effect is achieved because ERFE-based dose adjustments can be made at a much earlier stage during treatment compared to HGB-based dose adjustments. In humans, ERFE induction by rHuEPO occurs within 24 h,^35^ although earlier time points were not observed. In contrast, the HGB response to rHuEPO takes at least 2 weeks. This delay might lead to postponed rHuEPO dose reduction and, consequently, a more severe overshoot of HGB or unnecessary rHuEPO exposure for hyporesponders. Indeed, we found a longer duration of HGB overshooting using HGB-based dosing in comparison to ERFE-based dosing in CKD rats. Clinically, higher rHuEPO doses and HGB levels have been associated with adverse outcomes in CKD patients, even though the underlying mechanisms remain elusive.^54,55^ Therefore, utilizing ERFE-based rHuEPO dose titration could potentially prevent these adverse effects by enabling timely dose adjustments and facilitating switching to alternative treatments for hyporesponders. Future clinical trials are warranted to investigate the ERFE–HGB relationship in anemic patients and to assess the cutoff value of ERFE for predicting rHuEPO hyporesponsiveness. It is expected that using ERFE-based dose adjustment and identification of hyporesponsiveness during an early stage of rHuEPO treatment will not only enhance treatment efficacy but also lead to more cost-effective utilization of rHuEPO.

Our proof-of-concept study suggests that maximum induction of ERFE is a promising early biomarker for rHuEPO efficacy, useful to guide dose titration and patient selection in clinical practice. We propose a quantitative framework that can be integrated with biomarker research to enable a model-informed precision dosing strategy for anemia treatment.

## Data Availability

All underlying data are available in the article itself and its Supporting Information.

## Supporting Information Available

Detailed study design; the time course of hematological parameters in healthy controls versus anemic controls; the goodness-of-fit plots for PK/PD models; simulated time course of P3 in CIA rats; and parameters for the PK model (PDF)

## Abbreviations

ESAs: erythropoiesis-stimulating agents
rHuEPO: recombinant human erythropoietin
CKD: chronic kidney disease-associated anemia
CIA: chemotherapy-induced anemia
HGB: hemoglobin
ERFE: erythroferrone
PK/PD: pharmacokinetic/pharmacodynamic
BFU-Es: burst-forming unit erythroid cells
CFU-Es: colony-forming unit erythroid cells
EBs: erythroblasts (i.e., Pro-, Baso-, Poly-, and Ortho-EBs)
RET: reticulocytes
RBC: red blood cell
ADME: absorption, distribution, metabolism, and excretion
