# Correction to “Mechanism-Based Pharmacokinetic/Pharmacodynamic Modeling of Erythroferrone in Anemic Rats with Chronic Kidney Disease and Chemotherapy-Induced Anemia: An Early Biomarker for Hemoglobin Response and rHuEPO Hyporesponsiveness”

**DOI:** 10.1021/acsptsci.5c00174

**Published:** 2025-03-21

**Authors:** Lin Zhang, Peng Xu, Xiaoyu Yan

**Affiliations:** † Guangdong-Hong Kong-Macao Joint Laboratory for New Drug Screening, School of Pharmacy, 26451 The Chinese University of Hong Kong, Shatin 999077, Hong Kong SAR, P. R. China; ‡ Phase I Clinical Trial Center, Taihe Hospital, Hubei University of Medicine, Shiyan 442000, China

An additional affiliation for Peng Xu has been added: Phase I Clinical Trial Center, Taihe Hospital, Hubei University of Medicine, Shiyan 442000, China.

