# Supplementary material for: Mechanism-Based Pharmacokinetic/Pharmacodynamic Modeling of Erythroferrone in Anemic Rats with Chronic Kidney Disease and Chemotherapy-Induced Anemia: An Early Biomarker for Hemoglobin Response and rHuEPO Hyporesponsiveness
Source: ACS Pharmacol Transl Sci. 2024 Dec 11;8(1):189–202. doi: 10.1021/acsptsci.4c00575 (PMC11729431; doi:10.1021/acsptsci.4c00575)
Supplement: Supplementary file 1 — pt4c00575_si_001.pdf [file pt4c00575_si_001.pdf]

Supporting Information

Mechanism-based Pharmacokinetic/Pharmacodynamic Modeling of Erythroferrone in Anemic Rats with Chronic Kidney Disease and Chemotherapy-Induced Anemia: An Early Biomarker for Hemoglobin Response and rHuEPO Hyporesponsiveness

Lin ZHANG[1], Peng XU[1], Xiaoyu Yan[1*].

[1]Guangdong-Hong Kong-Macao Joint Laboratory for New Drug Screening, School of Pharmacy, The Chinese University of Hong Kong, Hong Kong SAR, P. R. China

***Corresponding author**: Xiaoyu Yan, PhD (Email: xiaoyuyan@cuhk.edu.hk)

Figure S1 The experimental timeline for experiments performed in CKD rats and CIA rats. Two weeks after first adenine administration, rats were stabilized for one week and screened for CKD development. CKD rats were either treated with rHuEPO (450 IU/kg, n=6; 1350 IU/kg, n=6) or saline (n=5). RBC and HGB were measured at baseline (Week 0, after 3 weeks feeding) and weekly after rHuEPO treatment. ERFE was measured within one day after the first injection of rHuEPO or saline. CIA in rats was induced by carboplatin injection. After one week ("Week 0"), CIA rats were allocated to the treatment groups receiving a single dose of rHuEPO (450 IU/kg, n=9; 1350 IU/kg, n=9) or non-treatment group (n=9) receiving saline only. Hematological parameters were measured at Week -1 (after carboplatin administration) and followed for three weeks after rHuEPO treatment. ERFE was measured within one day after rHuEPO or saline administration.

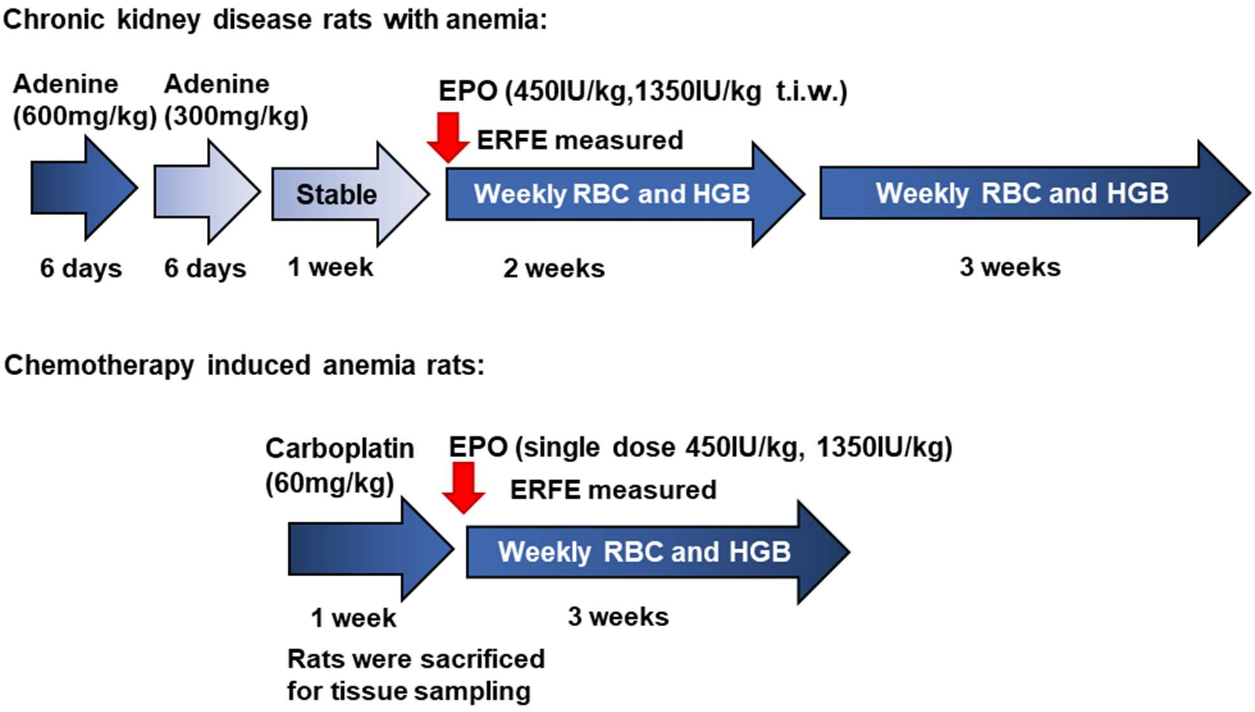

Figure S2 The experimental timeline for EPO-sparing effect study in CKD rats. CKD rats were dosed with an initial dose of rHuEPO at 100 IU/kg (tiw, i.v. injection) in "Week 0" and individualized for the maintenance period. ERFE was measured within one day after the 1st and 4th injection of rHuEPO or saline. Weekly erythropoietic responses (HGB and RBC) were followed for one month after the initial rHuEPO dose. The rHuEPO dose titration in Week 1 was based on the change of HGB (ΔHGB) in "Week 0" or the change of ERFE (ΔERFE) after the 1st injection of rHuEPO. The rHuEPO dose titration in Week 2 was based on the change of HGB (ΔHGB) in "Week 1" or the change of ERFE (ΔERFE) after the 4th injection of rHuEPO. The flowchart illustrates the inclusion criteria for CKD rats and dose titration rules of rHuEPO.

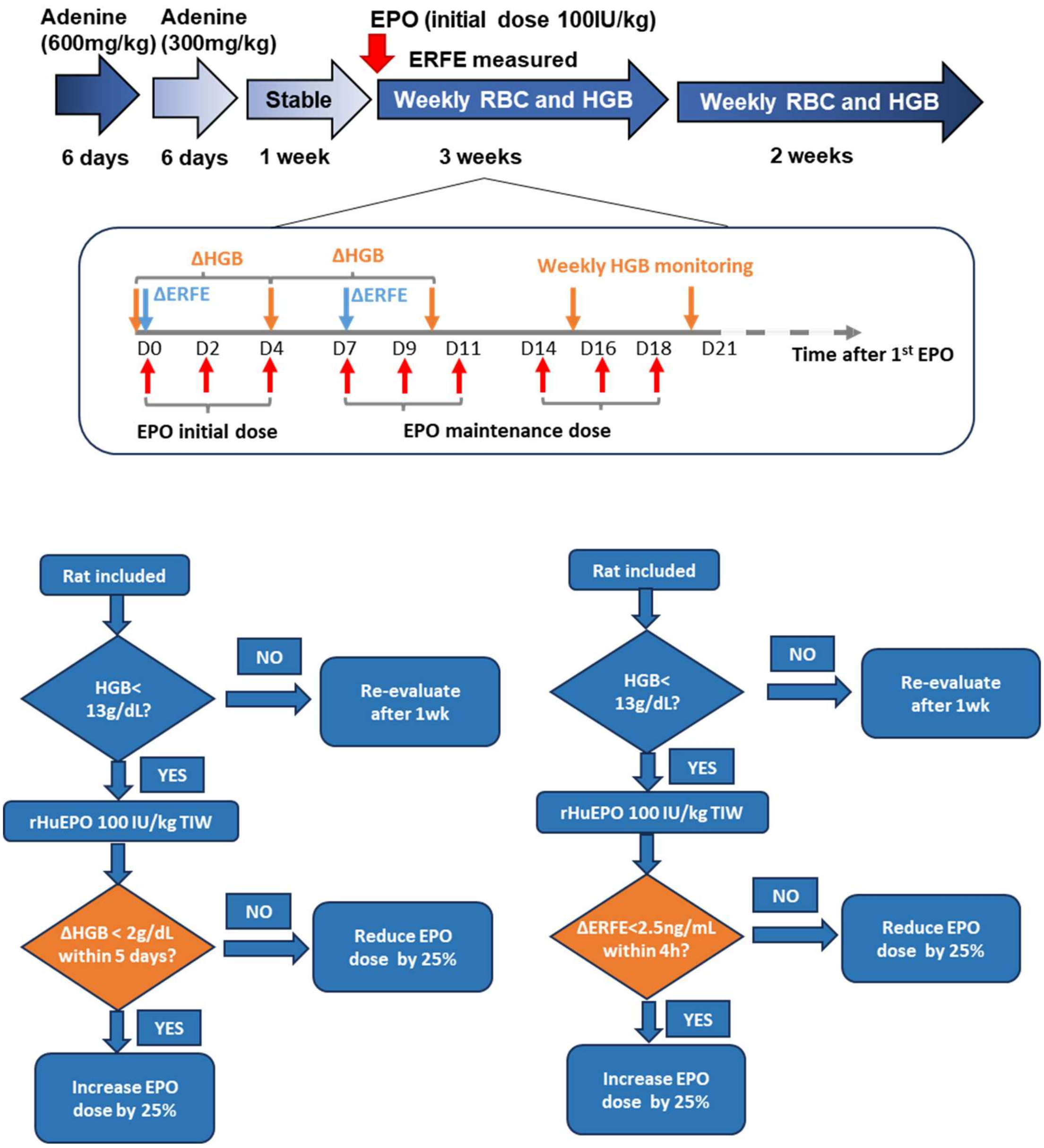

Figure S3 The time course of hematological parameters (HGB and RBC) in CKD control rats (n=5) versus healthy control rats (n=7) (A-B) and in CIA control rats (n=9) versus healthy control rats (n=7) after carboplatin injection (C-D).

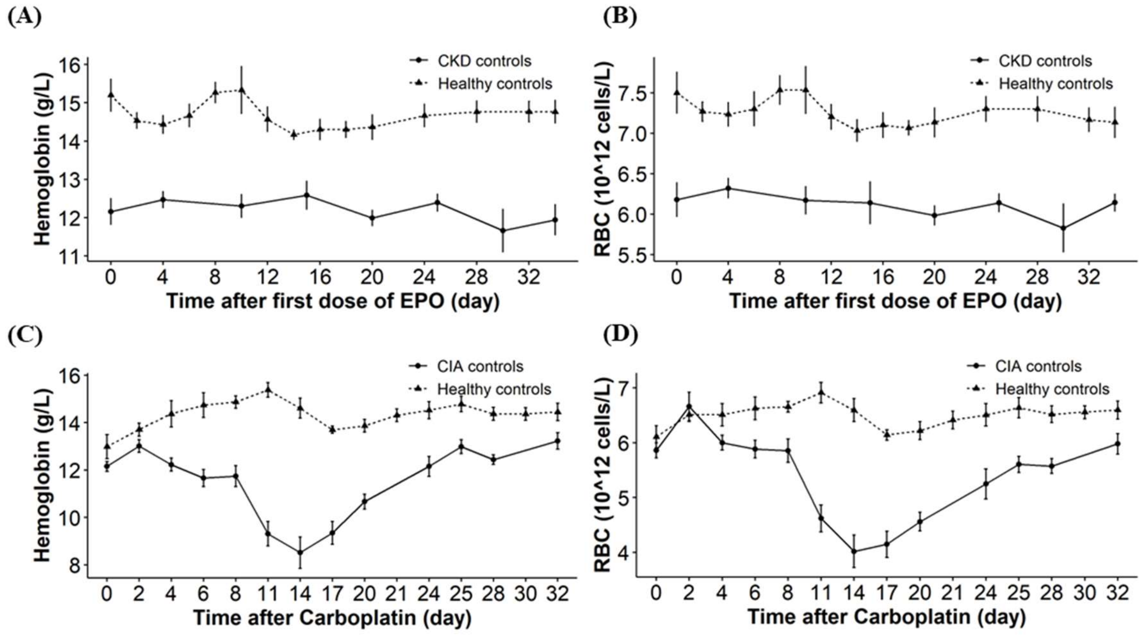

Figure S4 The goodness-of-fit plots for the ERFE, HGB and RBC model of CKD rats. The plots show the population predictions (A, E, I) and individual predictions (B, F, J) versus observations. Population predictions (C, G, K) and time (D, H, L) versus conditional weighted residuals. Straight lines in the top panels represent lines of identity. Straight lines in the lower panels represent zero lines.

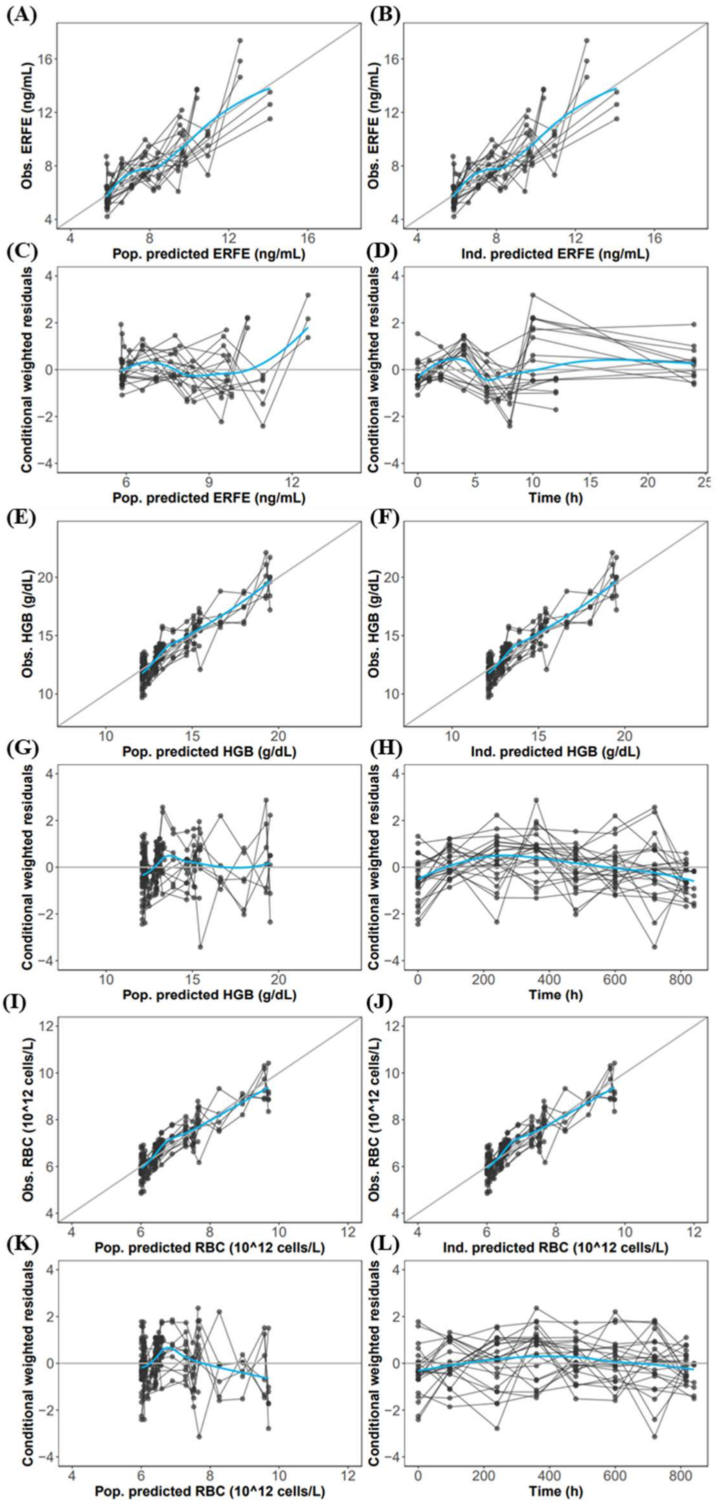

Figure S5 The goodness-of-fit plots for the ERFE, HGB and RBC model of CIA rats. The plots show the population predictions (A, E, I) and individual predictions (B, F, J) versus observations. Population predictions (C, G, K) and time (D, H, L) versus conditional weighted residuals. Straight lines in the top panels represent lines of identity. Straight lines in the lower panels represent zero lines.

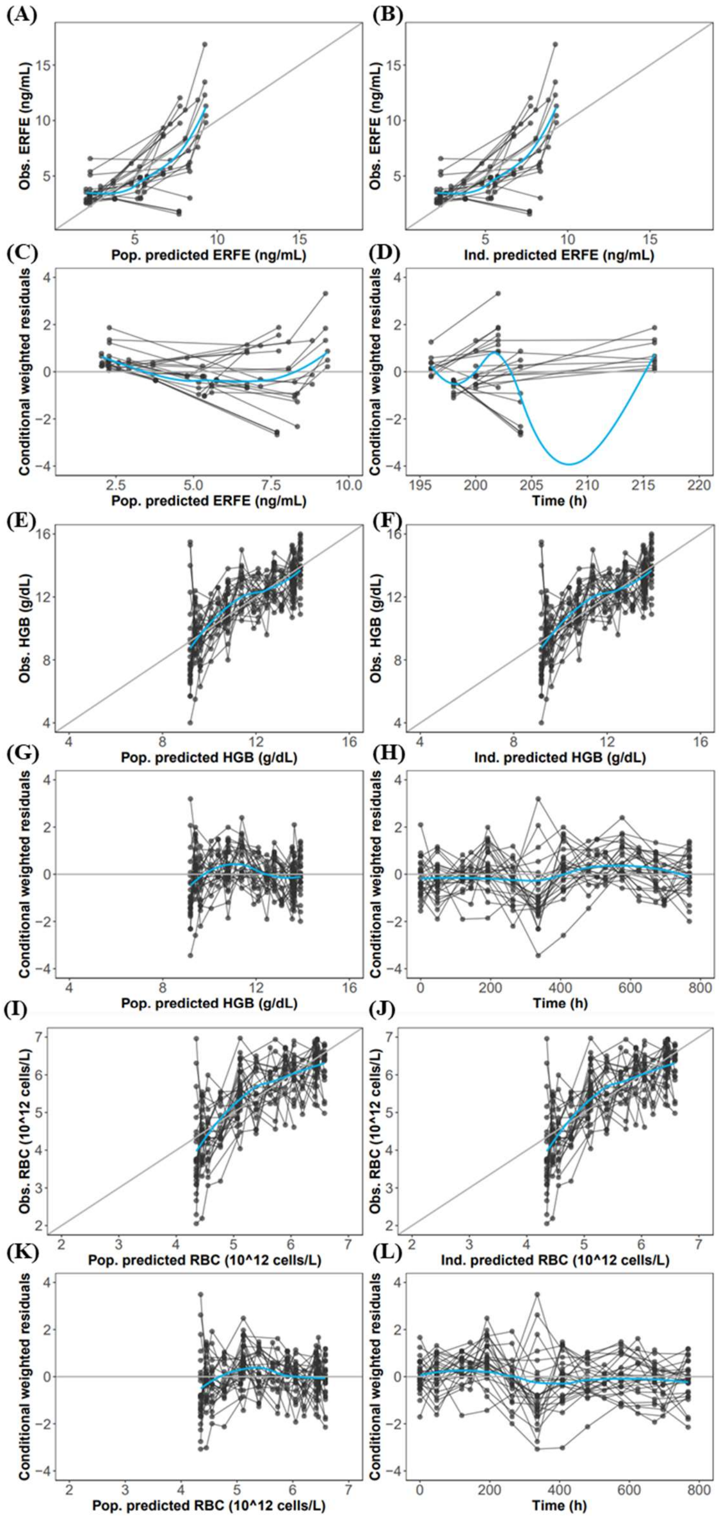

Figure S6 The simulated time course of P3 representing the major cell population producing ERFE in CIA rats. The arrow represents rHuEPO administration.

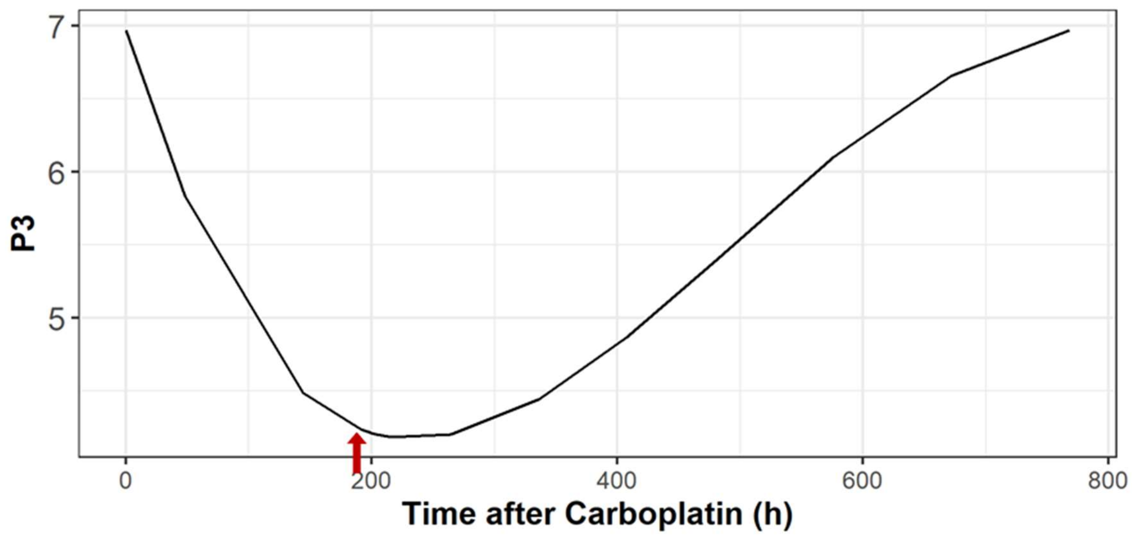

Table 1 Parameter estimates of the PK model

Parameter (unit)	Description	Estimate
Vmax ($mIU \cdot h^{-1} \cdot kg^{-1}$)	Michaelis-Menten capacity constant of rHuEPO	1993
Km ($mIU \cdot mL^{-1}$)	Michaelis-Menten affinity constant of rHuEPO	67.28
V_{EPO} ($mL \cdot kg^{-1}$)	Central volume of distribution of rHuEPO	61.18
Kel,EPO (h^{-1})	Linear elimination rate constant of rHuEPO	0.209
Kpt,EPO (h^{-1})	First-order intercompartmental rate constant of rHuEPO	0.171
Ktp,EPO (h^{-1})	First-order intercompartmental rate constant of rHuEPO	0.148
Vcarb ($mL \cdot kg^{-1}$)	Central volume of distribution of carboplatin	148.4
Kel,carb (h^{-1})	First-order elimination constant of carboplatin	2.788
K12 (h^{-1})	First-order intercompartmental rate constant of carboplatin	0.074
K21 (h^{-1})	First-order intercompartmental rate constant of carboplatin	0.425
K13 (h^{-1})	First-order intercompartmental rate constant of carboplatin	4.194
K31 (h^{-1})	First-order intercompartmental rate constant of carboplatin	5.611

Note: PK parameters were fixed based on previous studies.
